# Emerging Trends for Nonthermal Decontamination of Raw and Processed Meat: Ozonation, High-Hydrostatic Pressure and Cold Plasma

**DOI:** 10.3390/foods11152173

**Published:** 2022-07-22

**Authors:** Ume Roobab, James S. Chacha, Afeera Abida, Sidra Rashid, Ghulam Muhammad Madni, Jose Manuel Lorenzo, Xin-An Zeng, Rana Muhammad Aadil

**Affiliations:** 1 School of Food Science and Engineering, South China University of Technology, Guangzhou 510641, China; mahroba73@gmail.com (U.R.); james.chacha@sua.ac.tz (J.S.C.); 2 Overseas Expertise Introduction Center for Discipline Innovation of Food Nutrition and Human Health (111 Center), Guangzhou 510640, China; 3 Department of Food Science and Agroprocessing, School of Engineering and Technology, Sokoine University of Agriculture, P.O. Box 3006, Chuo Kikuu, Morogoro 14115, Tanzania; 4 National Institute of Food Science and Technology, University of Agriculture, Faisalabad 38000, Pakistan; afeeraabida162@gmail.com (A.A.); sidra87313@gmail.com (S.R.); raimadni0@gmail.com (G.M.M.); 5 Centro Tecnológico de la Carne de Galicia, Rúa Galicia Nº 4, Parque Tecnológico de Galicia, San Cibrao das Viñas, 32900 Ourense, Spain; 6 Área de Tecnología de los Alimentos, Facultad de Ciencias de Ourense, Universidade de Vigo, 32004 Ourense, Spain

**Keywords:** clean label foods, ozone, cold plasma, high-pressure processing, microbial safety, raw/frozen and processed meat products

## Abstract

Meat may contain natural, spoilage, and pathogenic microorganisms based on the origin and characteristics of its dietary matrix. Several decontamination substances are used during or after meat processing, which include chlorine, organic acids, inorganic phosphates, benzoates, propionates, bacteriocins, or oxidizers. Unfortunately, traditional decontamination methods are often problematic because of their adverse impact on the quality of the raw carcass or processed meat. The extended shelf-life of foods is a response to the pandemic trend, whereby consumers are more likely to choose durable products that can be stored for a longer period between visits to food stores. This includes changing purchasing habits from “just in time” products “for now” to “just in case” products, a trend that will not fade away with the end of the pandemic. To address these concerns, novel carcass-decontamination technologies, such as ozone, high-pressure processing and cold atmospheric plasma, together with active and clean label ingredients, have been investigated for their potential applications in the meat industry. Processing parameters, such as exposure time and processing intensity have been evaluated for each type of matrix to achieve the maximum reduction of spoilage microorganism counts without affecting the physicochemical, organoleptic, and functional characteristics of the meat products. Furthermore, combined impact (hurdle concept) was evaluated to enhance the understanding of decontamination efficiency without undesirable changes in the meat products. Most of these technologies are beneficial as they are cost-effective, chemical-free, eco-friendly, easy to use, and can treat foods in sealed packages, preventing the product from post-process contamination. Interestingly, their synergistic combination with other hurdle approaches can help to substitute the use of chemical food preservatives, which is an aspect that is currently quite desirable in the majority of consumers. Nonetheless, some of these techniques are difficult to store, requiring a large capital investment for their installation, while a lack of certification for industrial utilization is also problematic. In addition, most of them suffer from a lack of sufficient data regarding their mode of action for inactivating microorganisms and extending shelf-life stability, necessitating a need for further research in this area.

## 1. Introduction

Meat is high in protein, vitamins, and minerals, and it is one of the world’s most popular foods. Because of its intrinsic (nutrients, water availability, and pH) and extrinsic (transportation, processing, and storage) characteristics, meat is extremely susceptible to the development of pathogenic and spoilage microbes, for instance, *Campylobacter* spp., *Escherichia coli*, *Salmonella* spp., *Staphylococcus aureus*, lactic acid bacteria, and *Pseudomonas* spp. To guarantee food safety and conformity with quality requirements, all these microorganisms must be eliminated throughout industrial processing [1]. But in recent years, the safety of ready-to-eat (RTE) meats has been evaluated due to reported outbreaks that are associated with their consumption. During the repackaging of pasteurized meats, the core of the issue lies in post-process microbial contamination. Poultry and livestock producers can reduce the number of *Salmonella* (accounting for 31% of foodborne pathogenic deaths) that occur in animals before and during slaughter [2]. Besides, *Listeria* spp. grow during prolonged storage even at refrigeration temperatures (2–4 °C) [3]. This pathogen is sensitive to normal cooking, but it may contaminate the meat products after heating when exposed to the contaminated environment during cutting, slicing, and repackaging [4]. *L. monocytogenes* is of particular concern to meat and poultry products because it can grow in both raw and cooked meat [3]. This psychotropic Gram-positive pathogen causes a severe invasive disease called listeriosis. *L. monocytogenes* not only survive under a wide range of temperatures (1–45 °C) and pH (4.3–9.4), but it can also grow with water activity to a value of 0.92 and above. Furthermore, it can tolerate undesirable environmental conditions such as low-oxygen conditions, nitrite, and high salt content [3]. Food industries are putting efforts towards minimizing such post-process contamination and growth of pathogens by developing hurdle technologies [5]. Similarly, *S. aureus* can survive heat treatments and again can contaminate meat after cooking. Besides, the pre-and postslaughtering sources of *S. aureus* contamination include feed, feces, feathers, air, scald water and defeathering machines [6]. *S. aureus* has become a threat to public health because it can easily adapt to become methicillin-resistant *S. aureus* (MRSA), even during selective antimicrobial pressure, consequently causing staphylococcal foodborne illness that may lead to MRSA infection [7]. This opportunistic pathogen can grow in a wide range of temperatures, pH, and sodium chloride concentrations of up to 15% [8]. Raw and processed meat are the major food sources associated with food poisoning caused by *S. aureus*. The conventional techniques to evaluate the microbial safety of meat (i.e., culturing and biochemical testing) are time-consuming and labor-intensive [8].

Other thermal processing methods such as hot water and steam pasteurization [9], and chemical methods, for instance, lactic acid and sodium benzoate [10], trisodium phosphate and sodium hypochlorite [11], potassium sorbate [12], chlorine dioxide, and peroxyacetic acid [13], have been applied to reduce the bacterial counts in meat. For instance, Manzoor et al. [14] evaluated the effect of lactic acid spray (2–4%) on the microflora and shelf-life of buffalo meat displayed under modified atmospheric packaging. The aerobic plate count of sprayed carcasses and steaks was significantly lower than the unsprayed controls. Similarly, the bactericidal activity of lactic acid, levulinic acid, and sodium dodecyl sulfate was determined individually and in combination against Shiga toxin-producing *E. coli* (STEC) in pure culture conditions [15]. Results showed that the use of 3% lactic acid for 2 min in pure cultures reduced *E. coli* O26: H11, O45: H2, O111: H8, O103: H2, O121: H2, O145: NM, and O157: H7 populations by 2.1, 0.4, 0.3, 1.4, 0.3, 2.1, and 1.7 log CFU/mL, respectively. While the treatments of 0.5% levulinic acid, plus 0.05% sodium dodecyl sulfate, for less than 1 min reduced the populations of all STEC strains to undetectable levels [15]. In general, lactic acid concentrations less than 5% have not proven to be effective against *Campylobacter* in the form of a spray wash [16,17], even though levels of just 2% produced significant *Salmonella* reduction compared to other treatments [18]. The increased levels of to up to 8% caused considerable deterioration of the appearance of the carcasses, although the use of high acid concentration was beneficial for reducing the numbers of *Campylobacter* [16]. Changes in the texture and nutritional components may occur in meat owing to such processes [19,20]. In addition, chemical residues on meat surfaces cause health problems [21]. In the past, the poultry industry utilized 0.5–1 ppm chlorine and ice with a circulation system to lower chicken carcass temperature and bacterial load in the gizzard and intestine during the chilling process. This approach, however, may create cross-contamination in chiller tanks due to cycled poultry water. Chlorine and organic materials may react to generate halogenated organic compounds like chloroform, which relates to bladder and rectal cancer in humans. While considering the limitations and health concerns of chemical antimicrobial agents, it is necessary to seek other disinfectants or nonthermal technologies, such as ozone [22], high-hydrostatic pressure (HHP) [23,24], and cold plasma (CP) [25], as alternatives. Ozone gas, for instance, is one of the most potent oxidants known (for its use as a bactericide) because it can attack the cellular membrane of bacterial cells, leading to the lysis of cell structure and damage of DNA and proteins [26]. On the other hand, HHP, as a food preservation technology used for short-term treatment under high pressure, replaced the utilization of chemical preservatives or high temperatures [27]. Similarly, CP had been identified as a potential source of nitrite and its application in the meat industry as plasma-activated water is a great and efficient way of meat curing [28]. This review covers these technologies and their recent applications as nonthermal decontamination approaches for various meat products. 

## 2. Nonthermal Decontamination Technologies

### 2.1. Ozonation

In recent years, ozone (a naturally occurring water-soluble triatomic gas that can act as a strong oxidizing agent) has been of great interest to the processing industry. Bacterial inactivation through cell wall disruption, or lysis by ozone, is faster than other disinfectants that require time to invade the cell membrane [29]. It is, therefore, a very effective germicide against viruses, bacteria, and spores. The two mechanisms of inactivation include: (i) sulfhydryl group and amino acids of enzymes, proteins, and peptides oxidized to smaller peptides and (ii) polyunsaturated fatty acids oxidized to acid peroxides, resulting in cell death [30]. The effect of ozone treatment operating conditions on several microorganisms’ reduction is presented in Table 1.

**Figure 1:**
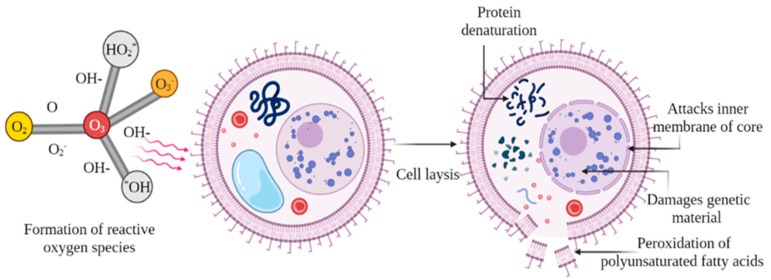
Schematic presentation of decontamination using ozone.

The significant oxidative properties of ozone justify its use as a decontaminating agent as an alternative to conventional agents (50% more effective than chlorine) [34]. It is highly efficient in killing viruses, bacteria, and protozoa within a short contact time. Figure 1 shows the action mechanism of ozone imparting decontamination activity. Ozone has an oxidative potential of 2.07 V, which is nearly double the oxidizing potential of chlorine (1.36) and greater than the efficacy of peroxyacetic acid (1.81) [34]. The exclusion of heat generation during ozone treatment makes it adaptable for heat-sensitive foods [35]. The threshold limit of ozone exposure has usually been calculated as 8 h/day at 0.1 ppm (0.2 mg/m^3^). However, its oxidizing power may prove toxic for humans depending upon the exposure length and level of concentration (0.1–0.3 ppm) [29]. Since all the consumer demands are fulfilled by ozone treatment, it can therefore be regarded as a “greener” additive. Furthermore, no specific guidelines for foodstuff related to the dosage of ozone are given, and it can thus be used in compliance with current industry standards of good manufacturing practice [36].

Commercially, ozone is applied for industrial waste deodorization and drinking water disinfection. However, its food application has increased since 1997, when the Food and Drug Administration (FDA) designated it as generally recognized as safe (GRAS). In December 2001, the USDA’s Food Safety Inspection Service (FSIS) approved ozone as a suitable and safe ingredient used for the treatment of ready-to-eat (RTE) meat and poultry products just before packaging [37]. Ozonation safely oxidized the contaminants without affecting their quality and left no residues behind [38]. It is an ecofriendly approach to disinfecting a wide range of materials and replacing other chemical disinfectants, such as chlorine, salts, and acids [33]. Although many researchers have proposed that ozonized water effectively improved the chemical properties and safety of meat, there are, however, no specific guidelines for its usage [32].

Gaseous ozone provides an advantage over aqueous ozone by invading pathogens residing in inaccessible places [39]. According to Giménez et al. [33], the gaseous ozone pulses (duration ranging between 5 and 10 min) effectively control microbial flora in beef every 30 min for 5 h using 280 mg/m^3^, whereby these treatments enacted the reduction (of >1 log) of LAB, mesophilic, and Enterobacteriaceae. Furthermore, this reduced the inoculated *L. monocytogenes* (102 CFU g/tissue) to below the detection limit and restricted its growth for 16 days at 4 °C. However, ozone treatment intensities of >58.66 mg/min in beef samples with a concentration of 286 mg/m^3^ are harmful concerning lipid oxidation and surface discoloration. Similarly, more than 10 min of exposure results in rancidity and color loss. In addition, ozone is a nonradical derivative of ROS (reactive oxygen species), which initiates oxidation reaction in foods. The production of free radicals is closely coupled to myoglobin oxidation. Similar results were previously demonstrated by Muhlisin et al. [31].

In chicken and duck breast, gaseous ozone (10 × 10^−6^ kg O_3_/m^3^/h) suppressed coliforms, aerobic, and anaerobic bacteria effectively. However, oxidation by ozone action led to the irreversible damage of cellular proteins and fatty acids in the cell membrane [31]. In addition, continuous exposure to ozone gas might increase oxygen generation due to ozone degradation. Chicken breast meat showed acceptable thiobarbituric acid reactive substances (TBARS) values until up to 3 days, while duck meat TBARS values increased with undesirable browning. According to the authors, ozone and other ROS are powerful oxidants that induce myoglobin and lipid oxidation. Metmyoglobin is produced as a result of myoglobin oxidation, which leads to meat discoloration, i.e., lower redness. Furthermore, ozone oxidizing activity increases rancidity and modifies surface color, affecting red meat quality [31]. Ozone can decontaminate and protect meat surfaces against microbial spoilage. For instance, turkey breast meat treated with ozone (1 × 10^−2^ kg/m^3^, for up to 8 h) reduced 2.9, 2.3 and 1.9 log CFU/g of total aerobic mesophilic bacteria, *Enterobacteriaceae*, and yeast and mold, respectively [32]. Furthermore, the increased ozone treatment time enhanced the number of carbonyls, as well as the cooking yield and water-holding capacity, of turkey samples. It can be assumed that after ozonation, structural changes of protein increased both of these properties owing to the amount of water stored in both cooked and raw meat, as this is closely related to the proteinaceous substances in tissues. Probably, a thin layer forms on the meat surface with this restricting water loss due to the protein denaturation caused by pH reduction. In addition, a partially denatured protein film layer (rich in connective tissue) could result in lighter colors on meat surfaces. Recent trends in packaging showed a delay in meat spoilage that involves the combination of nonthermal treatment with vacuum or modified atmospheric packaging using the plastic materials alone or in combination. Gertzou et al. [29] used 2–10 mg/L ozone to treat fresh vacuum packaged chicken legs for 16 days at 4 °C. According to the authors, the lower concentration of ozone (5 or 10 mg/L) for 1 h resulted in a 0.5–1.0 log reduction of *Pseudomonas* and a total viable count when combined with vacuum packaging, whereas an increase in the intensity of gaseous ozone up to 10 mg/L resulted in >1.0 log cycles to the population of *Enterobacteriaceae*, lactic acid bacteria, and yeast and molds. Moreover, the shelf-life of vacuum-packaged ozonated chicken legs was extended to 6 days in comparison to single vacuum packaging. However, the physicochemical parameters noticeably varied depending on the intensity of the ozonation and storage period. In contrast, Zouaghi et al. [30] investigated that 0.6 ppm for 10 min was the best ozonation condition for maintaining the acceptable color, texture, and sensory quality of dried chicken breast fillets stored in modified atmosphere packaging with 80% N_2_ and 20% CO_2_ gas combination at room temperature.

Cantalejo et al. [22] used the hurdle approach to preserve raw meat products by combining ozone and freeze-drying. However, the microorganisms’ growth ceased for a longer period in a well-lyophilized product due to lower water activity and residual humidity (<10%). Ozone treatment (0.6 ppm for 10 min) combined with lyophilization reduced total aerobic mesophilic bacteria (6.8 log CFU/g) and lactic acid bacteria counts (4.77 log CFU/g) with an extended shelf life of 8 months. Nevertheless, increasing ozone treatment intensity (concentration and time) decreased the aerobic mesophilic counts significantly. In contrast, four-month shelf-life stability was obtained for the lyophilized samples (alone). Furthermore, 0.4 ppm ozonation showed a negative effect on the chicken meat sample by increasing both chewiness and hardness, while lyophilized samples were susceptible to oxidation when stored in undesirable conditions, producing unwanted organoleptic characteristics [22]. These findings introduced the need for a suitable packaging hurdle for ozonated freeze-dried samples.

The innovative nonthermal ozonation method is beneficial as it is cost-effective, chemical-free, and eco-friendly, as well as easy to use. However, ozone application in the meat industry is challenging because of its strong oxidative power, which might cause damage to the meat’s cellular proteins and fatty acids. Moreover, ozone is quite unstable, with even exposure to light potentially degrading it; hence, it cannot be stored [40]. Furthermore, ozone requires on-site generation, thus cutting the cost of control of chemical production. Ozone is water-insoluble; special mixers are therefore required to solubilize it, which also limits ozone application for the surface disinfection of fresh fruit and packaged food compared to microbial inactivation within the food samples. Furthermore, in comparison to other disinfection processes, the installation of ozone technology is highly complicated and demands a large capital investment. All these disadvantages limit ozone application in food industries. For that reason, further research is needed to overcome these limitations, as well as expand ozone technology utilization in the food industry.

### 2.2. High Hydrostatic Pressure (HHP)

HHP is a major trend in the food industry nowadays in terms of clean label technology. It is the most modern method of increasing the shelf stability of food products [41,42]. HHP is a response to the challenges faced by the industry and provides a competitive advantage, which is undoubtedly worth implementing sooner rather than later. According to Lee et al. [43], global revenues from the high-pressure food protection (i.e., HHP) market amounted to USD 1055 million in 2019 and will reach USD 2123 million in 2025, with a compound annual growth rate of 12.34% from 2021–2025. HHP can achieve food safety, inactivate pathogens, such as *Salmonella*, *Listeria*, and *E. coli*, and prevent recontamination, seeing as the packed product is virtually impossible to recontaminate. HHP reduces microorganisms or eliminates them and/or reduces chemical preservatives. Table 2 summarizes the range of parameters used in HHP to decontaminate meat and meat products. In general, HHP (a single step at 86,000 psi for 3 min) as a clean label (no preservatives) technology was able to effectively double the shelf-life of meat products, with the control product lasting for about 30 days compared to 60 days for the HHP product, concerning pathogen control.

Although the microbiological quality of poultry meat depends on several critical factors such as the physiological status of an animal, temperature, and other conditions during slaughter, HPP (for 10 min at 500 MPa) inhibited *Salmonella* ser. *Enteritidis* during 12 °C storage (0.48 log CFU/g) and extended the shelf-life of the chicken meat by 6 to 12 days [44]. Moreover, the population of *Salmonella* ser. *Enteritidis* remained below or near the detection limit during storage at 4 °C. According to the authors, the inactivation of *Salmonella* in HHP-treated samples was highly related to the product (raw material), as well as to the strains of *Salmonella* being inoculated. As compared to the control samples, HHP-treated samples showed unpredictable changes in the distribution and survival of the *Salmonella* strains at different inoculum levels and storage temperatures [44]. These results highlighted a potential mechanism involving the ecological modification of the food microbiota via different treatment conditions, which is crucial for designing and applying a new or different technology in the food industry.

HHP (applied for 5 min at 400 MPa and 1 min at 500 MPa) not only lowered *Salmonella* spp. (>3 log units) populations in frozen fillets of chicken but also improved the color and texture profile, as compared to the control samples [45]. However, HHP at increased pressure (600 MPa) flattened and deformed the cells while increasing the holding times (5 min) and elongating the cellular tissues. Although changes in the textural profile of meat depend on the protein system, rigor state, and processing parameters (i.e., temperature, pressure level, and time), researchers have observed an increase in firmness and work area for the HHP-treated cooked chicken breast fillets, as compared to the control [45]. Additionally, no significant differences were found between *Salmonella* spp. counts for pressurized samples treated for 1 and 3 min and among the treatments of 5, 7 and 9 min, which indicated the fact that HHP treatments quickly destroy sensitive cells, while the remaining cells produce stress adaptation and higher resistance [45].

The surface and interior of HHP-treated ham (applied for 10 min at 450 MPa or 5 min at 600 MPa) depicted a 2 and 3 log units reduction in *L. monocytogenes* populations, respectively, despite the varying levels of water activity [66]. However, authors observed that the effectiveness of HHP treatments (at 600 MPa for 5 min) against *L. monocytogenes* was influenced by the physicochemical properties of the food matrix, such as lower water activity, which diminished the antimicrobial impact of HHP. The microbial groups which are adaptive to low water activity and high salt content will survive during the ripening of dry-cured ham. According to the authors, low water activity in dry-cured ham can shield microorganisms and reduce the decontamination efficacy of HHP [66]. In addition, the HHP treatment of fresh ground chicken meat for 10 min at 350 MPa, in conjugation with 0.75% carvacrol (essential oil extract), inactivated *L. monocytogenes* and *Salmonella* to 1 log unit at 4 °C [46]. Furthermore, selective, and nonselective agar plate counts were compared to examine HPP-induced bacterial injury and its recovery, which showed significant results for the counts of HPP-treated *Salmonella* (i.e., 1–2 log difference was found on different growth media). On the other hand, carvacrol inhibited the growth and recovery of the HPP-treated bacterial cells [46].

According to Jiang and Xiong [68], the synergistic effect of antimicrobial agents with postpackage pasteurization by HHP was an effective and economic approach. The combination of HHP (applied for 10 min at 400 MPa and 17 °C) and active packaging technologies (antimicrobial films), along with cold storage (6 °C) against *L. monocytogenes* strains in cooked ham, was a successful alternative to conventional thermal treatments [67]. It should be noted that the intensity of treatment had a great effect on the treatment’s success. In terms of microbial inactivation, *L. monocytogenes* had recovered from the damage induced by 400 MPa for 20 min during cold storage in dry-cured ham. The damage that occurred to the membrane was, however, significant at 600 MPa for 5 min [69].

Similarly, HHP at 500 MPa for 10 min reduced *Pseudomonads*, *S. Enteritidis*, *Brochothrix thermosphacta*, *Enterobacteriaceae*, lactic acid bacteria, and yeast/mold populations during various storage conditions (4–12 °C) for 2–6 days [44]. In emulsion-type sausages (supplemented with 1% vinegar stored for 5 weeks), 500 MPa HHP treatment for 12 min for a clean-label substitute of NaNO_2_ reduced *C. perfringens* vegetative cells and spores by 4.8 logs and 2.8 log, respectively [64].

Furthermore, combining biopreservation technologies with high pressures can substitute the use of food preservatives. For instance, a mild HHP (for 5 min at 300 MPa and 10 °C), lactic acid bacteria (*Pediococcus acidilactici*, HA-6111-2) and bacteriocin (bacHA-6111-2) combination on traditional Portuguese RTE sausage (*Chouriço de carne*), extended the refrigerated storage by controlling the food microbiota [65]. Results showed an increased lower cell count (~0.4 log CFU/g) in HHP slices. According to the authors, fat content can act as a baroprotective for microbial cells. However, pressure can also disrupt fat globules, which release more viable cells and more bacterial cells, becoming visible on the plate media [65]. HHP with bacteriocin technology was used to enhance the already applied hurdles (e.g., high salt concentration) to help manufacturers control the microbiota during extended refrigerated storage. A few minutes of exposure to a pressure of up to 6000 bars is sufficient to inactivate bacteria (including *E. coli* and Salmonella), mold, fungi, and other dangerous pathogens, while not interfering with the structure of the product itself and eliminating the need to add unnecessary preservatives. For instance, HHP treatment (for 5–10 min at 100–300 MPa) of chicken breast meat reduced *E. coli* 3014, *Salmonella* spp. 3064, and *L. monocytogenes* ATTC 23,074 populations by 3.0–5.3 log units [70].

### 2.3. Cold Plasma (CP) Technology

**Figure 2:**
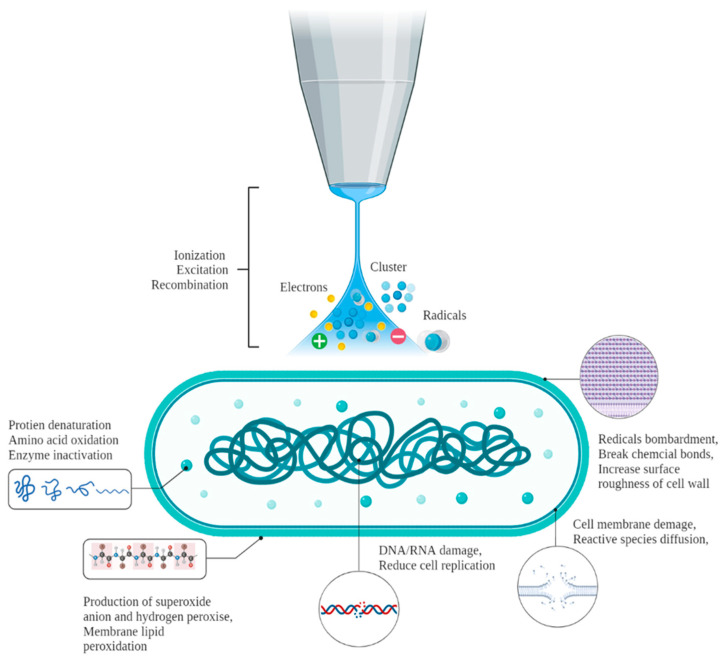
Effect of CP sterilization on a bacterial cell.

CP is among the emerging decontamination technologies that preserve food at ambient or sublethal temperatures and is being extensively explored for application in raw and processed meat products. CP is produced by exposing gas (air or any gas mixture) between electrodes to generate a very high strength of an electric field by using dielectric barrier discharge (DBD), radio frequency, and microwave power sources. The ionized gases consist of chemically reactive species, such as positive and negative ions, radicals, electrons, excited and neutral molecules, ultraviolet photons, and visible light [71]. Chemical bonds are split by these antimicrobial active species, with charged particles present in the biomaterials, which initiate various biochemical reactions leading to the death of microorganisms. In particular, reactive nitrogen species (RNS) and reactive oxygen species (ROS), along with hydroxyl radicals, ozone, singlet oxygen, hydrogen peroxide, superoxide anion, hydroperoxyl, nitrogen dioxide radical, nitric oxide, peroxyl, alkoxyl, alkylperoxynitrite, peroxynitrous acid, and peroxynitrite are active antimicrobial agents that injure or kill microbes during direct contact via reacting with various macromolecules present in the bacterial cells [72]. Figure 2 shows a schematic representation of the cold plasma sterilization of bacterial cells.

Additionally, in the bacterial cell wall, the colliding reactive species react with lipopolysaccharide and peptidoglycan, which breaks chemical bonds and reacts with intracellular substances by entering the cytoplasm [72]. Consequently, it induces DNA damage, photodesorption, and lipid peroxidation. According to Chaplot et al. [73], atmospheric CP reduced 5.3 log CFU/cm^2^ *Salmonella typhimurium* in poultry meat. Similarly, Choi et al. [74] reported a 1.5 log reduction of *E. coli* O157: H7 and more than a 1 log reduction in *L. monocytogenes* of pork after CP jet treatments (20 kV for 0–120 s). Stratakos et al. [75] achieved 0.9 and 1.82 log CFU/cm^2^ reductions of *E. coli* levels using CP jet (6 kV, 20 kHz, 99.5% helium, and 0.5% oxygen) after 2 and 5 min, respectively, in raw beef. Furthermore, the sub-lethally injured cells were unable to recover and eventually died during unfavorable cold storage conditions (4 °C). Similarly, Gök et al. [28] applied CP treatments (for 180 and 300s) to reduce *S. aureus* (from 5.78 to 0.85 log CFU/cm^2^), *L. monocytogenes* (5.71 to 0.83 log CFU/cm^2^), total aerobic mesophilic bacteria (1.41 log CFU/cm^2^), and yeast-mold counts (1.66 log CFU/cm^2^) in pastirma (dry-cured beef product) samples. CP treatments (at 1 MHz and 2–3 kV for 30–180 s) were applied to chicken skin and breast fillets and reduced *Campylobacter jejuni*, ranging from 0.78–2.55 log CFU/cm^2^ (using argon as feed gas) to 0.65–1.42 log CFU/cm^2^ (using air) [76]. According to the authors, ionized argon has a high electron density, thus generating more antimicrobial reactive species. Furthermore, increased exposure time significantly led to a higher reduction of *C. jejuni*. However, CP raised the temperature on the surface of the treated samples (i.e., 61 °C), which could be another reason for the higher decontamination. The effects of CP on different types of meat and meat products contaminated with microorganisms at different operating conditions are listed in Table 3.

The efficiency of a treatment depends on a variety of factors including CP device, microorganism composition and initial levels, and food type [92]. Sometimes, CP treatment activated a detoxification system, in which proteins interact with the CP-reactive species and have a decreased inactivation rate [93]. For instance, Benecke et al. [91] observed a 0.3 log reduction in *S. typhimurium*, whereas the bacterial counts of *L. monocytogenes* and *E. coli*. remained unchanged after CP treatment (0–120 s) in mortadella-type sausage slices. It was established that *S. typhimurium* was more sensitive to CP treatment than *E. coli*, and *L. monocytogenes*, being a Gram-positive bacterium, has a thicker outer membrane than Gram-negative *S. typhimurium*. CP reactive species can easily diffuse through the thinner cell wall of Gram-negative bacteria and possess stronger antimicrobial effects. Furthermore, it was revealed that 120 s of CP treatment reduced more *L. monocytogenes* (6.58–6.25 log CFU/g) and *E. coli* (5.63–5 log CFU/g) compared to a 30 s treatment over 21 days of storage. One suggestion was to use a higher power CP device for higher electron and ion density, as well as ultraviolet photons. Because high initial cell counts resulted in several clumps of bacteria, which then supposedly protected each other against CP treatments in synergy with the protein (13%) and high fat (20%) content of the mortadella-type sausage slices. The authors of [94] demonstrated the impact of atmospheric DBD-CP for inactivating four test strains (i.e., *S. aureus*, two *E. coli* strains, and *L. monocytogenes*) in vacuum-packaged and nonpackaged beef longissimus samples, over a 10-day vacuum period, and a subsequent 3-day period of storage in aerobic conditions. Results indicated a more than 2 log reduction in the bacteria without affecting the integrity of polyamide-polyethylene packaging film. Moreover, the low intensity of CP treatment works better to reduce bacterial numbers compared to high-power treatment.

The use of CP technology for the quality and safety of meat and meat products, including pork, chicken, and beef, is a recent concept [95]. This is due to the potential of CP to inactivate a broad range of microorganisms effectively, including biofilms, spores, and even some viruses in foods. Misra et al. [96] reviewed the greater attention of the meat industry towards the potential application of CP technology regarding effective decontamination techniques. CP has several benefits in poultry processing and can work with other hurdle approaches, such as controlled packaging [97]. This technology has a distinct approach to packaging, among the various possible configurations, which consists of ionized gas (air or a modified gas) and the food product in a sealed package being exposed to a strong electric field [98]. CP produces reactive species at high voltage (>10,000 kV) in the packages, having antimicrobial, fungicidal, and viricidal effects. For instance, UV-induced DNA modification and charged particles ruptured the bacterial cell membrane due to strong electrostatic forces [99].

Moutiq et al. [80] used atmospheric CP treatments (at 100 kV) on chicken breast samples to achieve a 2 log CFU/g reduction in natural microflora within 5 min of treatment. According to the authors, ROS and RNS were responsible for reducing the microbial count. Furthermore, the shelf-life study (24 h) exhibited 1.5, 1.4 and 0.5 log lower populations of mesophiles, psychotropic, and Enterobacteriaceae, respectively, in CP-treated samples in comparison to control samples. Similarly, Lee et al. [100] reported that the total aerobic bacterial count was reduced by 1.2 log CFU in chicken breasts when 5 min of plasma treatment was applied using a flexible thin layer DBD plasma set-up. In-package atmospheric DBD-CP had a great effect on the microbial safety of chicken breast meat. CP (at 70 kV) reduced more than 90% of the inhibition of psychrophilic and foodborne pathogens; however, when treatment time extended beyond 60 s, this resulted in overall paler breast meat [81]. According to the authors, electron-driven ionization and dissociation caused micro discharge and a breakdown of gas molecules and/or reactive radicals. In-package ozone could be another attribute for microbial inactivation, despite charged particles, ions, radicals, reactive gas species, and radiation [101].

The in-package CP system involves treatments within sealed packages, which prevents the product from post-process contamination. The composition of gas inside the package is another critical factor influencing the antimicrobial efficiency of CP treatment. In a study regarding ham, the authors of [89] concluded that modified atmosphere gas compositions, ham formulation, treatment time, and post-treatment storage (1 and 7 days at 4 °C) affect the reduction of a five-strain cocktail of *L. Monocytogenes*, along with causing changes in the quality of the ham subjected to in-package CP treatment. They achieved a 2 log (CFU/cm^2^) reduction in *L. monocytogenes* after treatment and a more than 6 log reduction after 7 days of post-treatment storage, regardless of ham composition. Similarly, chicken fillets were packaged in food trays (filled with modified gas or air) and exposed to in-package CP (80 kV for 180 s). Results indicated fewer microbial counts (mesophiles, psychrophiles, and *Pseudomonas* spp.) in the treated samples filled with modified gas (<3.5 log CFU/g) compared to the treated samples filled with air (>6 log CFU/g) and control samples (<4 log CFU/g). It was also established that psychrophiles (especially *Pseudomonas*) constituted major spoilage bacteria on the surface of fresh chicken meat in either air or high oxygen modified atmospheres during storage at 4 °C [78]. Furthermore, the ozone level was three times higher in the modified atmosphere than in the air. Besides, inside the packages, voltage increases from 55 kV to 80 kV caused increasing ozonation but had no effects on the microbial populations (mesophiles or psychrophiles).

Wang et al. [102] indicated that an increase in treatment time (>3 min) further enhanced the antimicrobial efficacy of CP-treated air-packed raw chicken meat. Zhuang et al. [82] recommended modified atmospheric packaging (containing 60% CO_2_ and 35% O_2_) before CP treatment (for 60 s at 60 kV) in chicken breast meat (under refrigerated storage). The results demonstrated an increase in ozone generation at 60 kV compared to 80 kV. This strengthens the hypothesis that the effect of oxygen content on ozone formation in CP packages depends on package configuration, voltage, and treatment time. It was also established that increasing the oxygen content (from 35 to 90%) resulted in increased psychrophilic counts (from 5.5 log to >7.0 log) on raw meat. However, increasing oxygen content (>35%) in packages or increasing CP treatment voltage might not always benefit the in-package CP-based antimicrobial treatments.

Recently, in-package DBD-CP at 24 kV for 3 min proved to be a promising nonthermal technology for inactivating natural mesophilic aerobic bacteria (0.7 log CFU/cube), *Salmonella* (1.4 log CFU/cube), and Tulane viruses (1.1 log PFU/cube) in chicken breast cubes [85]. Furthermore, the increasing voltage from 22 kV to 24 kV led to a noticeable increase in the *Salmonella* inactivation level. In addition, 4 °C post-treatment storage (21 days) reportedly enhanced the microbial inactivation efficacy (0.7–0.9 log CFU/cube) of CP treatment. According to Lee et al. [85], within closed containers, reactive species diffused into food tissues and controlled microbial growth during storage. During storage, hermetic sealing is involved, ensuring more substantial microbial growth inactivation in the packed food. Similarly, in-package DBD-CP treatment (39 kV for 3.5 min) reduced *E. coli* O157:H7 (3.9 log CFU/cube), *Salmonella* (3.7 log CFU/cube), *L. monocytogenes* (3.5 log CFU/cube), and Tulane virus (2.2 PFU/cube) in boiled chicken breast cubes [83].

Another study found that in-package atmospheric DBD-CP (at 220 V and 60 Hz for 0.3–2.5 min) caused a 1.7 log CFU/sample *Salmonella* reduction in a boiled chicken breast, while the D-value increased (from 0.2 to 1.3 min) with the increase in the initial inoculum concentration (from 3.8 to 5.7 log CFU/sample) [84]. In a similar study, high-density cells (pure culture) induced a protective effect, suggesting that *Salmonella* could be resistant to CP (for 1 and 3 min). Interestingly, with increasing CP treatment time (up to 6 min), the reduction of *Salmonella* increased [73]. However, the edible-film coating and shaking of the product induced better results in terms of uniform microbiological decontamination, color, and a smoother surface of chicken breast meat cubes in salad products [84].

The prolonged refrigerated storage of grounded meat increases lipid oxidation and microbial populations. Natural antioxidants such as essential oils are preferable for use in meat as an alternative to synthetic preservatives, like butylated hydroxyanisole, tertiary-butyl hydroquinone, and butylated hydroxytoluene. For instance, Gao et al. [86] used 1% rosemary (*Rosmarinus officinalis* L.) extract on in-package DBD-CP treated ground chicken breast patties. A post-treatment storage study revealed more pronounced microbial populations in the control samples (1.70 log cycles) than in the CP-treated samples for 180 s at 70 kV (1.14 log cycles). Moreover, the physiological profile of the bacterial population can be used as an efficacy indicator of the synergistic rosemary and CP treatment [103]. Similarly, the combination of essential oil marinades (*Crocus sativus* L., *Zataria multiflora Boiss*, and *Allium sativum* L.) and CP treatment not only reduces *S. aureus* (~>2 log CFU/g) and *E. coli* (4 log CFU/g), but also helped to maintain stable antimicrobial activities (over 14 days) in breast chicken fillets [79].

Lin et al. [71] preserved fresh poultry meat against the contamination of *S. typhimurium* by fabricating thyme essential oil/silk fibroin nanofibers (electrospun), and CP treatment was also introduced. CP modified the surface properties of the nanofibers and also enhanced the release of thyme essential oil from the nanofibers. The high antibacterial effect of this treatment reduced the number of *S. typhimurium* in duck and chicken meat by 6.06 and 6.1 log CFU/g, respectively, in comparison to the control group. This implied that the essential oil increased the electrical conductivity, which induced electrolyte leakage and led to cell membrane damage. Moreover, high-power CP combined with cold storage led to a population reduction of *S. typhimurium* of 1.85 log CFU/g, whereas *L. monocytogenes* was reduced by 2.55 log CFU/g, being near or below the detection limit.

On the other hand, the combined effect of peroxyacetic acid (200 ppm) and CP treatments reduced *Salmonella* populations in poultry meat. CP increased the concentration of reactive species such as H_2_O_2_, O, O_2_, O_3_, HO_2_, and OH, which imparted stronger antimicrobial action than the individual treatments. According to the authors, the dose-dependent peroxyacetic acid released active oxygen that disturbed the sensitive sulfur bonds and sulfhydryl within the cell membrane, or disrupted the chemiosmotic function of the cytoplasmic membrane, phospholipid bilayer, and the cell wall [73]. Similarly, a combined CP (0–28 kV for 180 s) and salt (1–3%) treatment reduced *L. innocua* by 1.75–1.51 log and 1.78–1.43 log (CFU/cm^2^) during post-treatment storage at 4 °C and 23 °C, respectively [90].

The direct application of CP to unpackaged food currently meets with obstacles, as it is not certified for industrial use as an antimicrobial technology [104]. CP treatments are also limited to sterilized foods because of irregular food shapes, restricted volumes, and different sizes [86]. Moreover, the impact of CP treatments on the physiology, microbiology, and toxicology or allergology of a food matrix remains unclear. There is also a lack of sufficient data on its mode of action to inactivate microorganisms and extend the shelf stability of food. Therefore, the in-package CP technology adaptation with efficient plasma sources requires further studies to develop, formulate, and preserve packaged food [83].

## 3. Conclusions

The changes caused by the COVID-19 pandemic have forced food producers to reorganize their processes, analyze their priorities, and redesign many products. Innovative technologies are more and more intensively used to provide the highest quality food products to the growing group of conscious and demanding consumers. Current meat disinfection procedures involve the use of various chemical antimicrobial agents. However, the stringency of food regulatory laws is increasing with the implementation of new performance standards for meat processing. Further, meat processors cannot depend upon a single intervention to guarantee the safety of meat products without risking their qualitative characteristics (discoloration, moisture loss, textural changes, and other changes in sensory attributes). It has been investigated and found that nonthermal processing technologies replace thermal processes by efficiently reducing microbial contamination, retaining nutrients and increasing quality. Ozonation, HHP, and CP are only a few of the technologies created for flexibility, power efficiency, economy, and sustainability. To lower meatborne bacterial pathogens, the suitable hurdle approach is that which combines multiple antimicrobial treatments. However, since these technologies suffer from pertinent drawbacks, further research and future investigation are still warranted. Research efforts need to be directed towards obtaining a clearer understanding of their mechanisms of action, as this will pave way for their applicability in various foods treatments and their synergistic incorporation in hurdle technologies. This is due to the fact that although these are clean label technologies, in most cases, depending on the food under treatment, they are not used singularly but alongside other techniques. Moreover, deliberate efforts ought to be carried out in working with national, regional, and global quality and regulatory agencies to ascertain the safety of the technologies for consumers. As much as the clean label technologies are preferred by consumers, some of them are still held as being in a gray area, as it is not yet clear whether they should be generally recognized as safe (GRAS). Furthermore, technological modifications from an equipment point of view are also a necessity to ensure that these technologies are not only cost-effective but that they also require a considerate and affordable investment scheme to be adopted and utilized by the small-scale food processors. This is due to the fact that these are not only the main processors of foods in many countries, especially in third-world countries, but proportionately, they are thought to be the ones available in huge numbers compared to the medium- and large-scale food processors.

## Figures and Tables

**Table 1:** Ozone applications to decontaminate meat and meat products.

Sample	Specification	Microbes	Highlights	Reference
Chicken legs	2–10 mg/L for 1 h combined with vacuum packaging (polyamide/polyethylene bags) stored at 4 °C for 16 days.	TVC, *Pseudomonas* spp., LAB, Yeast-molds, & *Enterobacteriaceae*	6-day shelf-life extension compared to vacuum packaging alone (4-day extension). Positively affected odor, texture, and taste retained an acceptable score for 14–16 days.	[29]
Chicken meat (freeze-dried)	0.6 ppm at 4 °C (90% RH) for 10 min.	TAMB, LAB, *E. coli*. & *Salmonella* spp.	1.1 log CFU/g was observed in TAMB and LAB. *E. coli*. and *Salmonella* spp. was not detected. Combination with MAP (20% CO_2_, 80% N_2_) improved the texture and sensory proprieties.	[30]
Chicken meat (freeze-dried)	0.4–0.7 ppm at 4 °C (90% RH) for 10–120 min.	LAB & TAMB	Reduced 4.77 and 6.8 log CFU/g, respectively. The combined use of ozone and lyophilization would be useful for extending shelf-life to 8 months.	[22]
Chicken breast meat	10 × 10^−6^ kg O_3_/m^3^/h for 3 days.	Coliform, aerobic, and anaerobic bacteria	Aerobic: 2.96 log CFU/g (untreated = 5.35 log CFU/g) Anaerobic: 2.18 log CFU/g (untreated = 4.63 log CFU/g) Coliform: 1.74 log CFU/g (untreated = 3.35 log CFU/g)	[31]
Duck breast meat			Aerobic: 2.52 log CFU/g (untreated = 4.11 log CFU/g) Anaerobic: 3.46 log CFU/g (untreated = 3.95 log CFU/g) Coliform: 1.39 (untreated = 3.28)	
Turkey breast meat	1 × 10^−2^ kg/m^3^ at 22 °C (21.6% RH) for 8 h.	TAMB, *Enterobacteriaceae* & yeast-mold	Reduced 2.9, 2.3 and 1.9 log CFU/g, respectively.	[32]
Beef (sliced)	218–286 mg/m^3^, 5–20 pulses for 2–40 min with intervals of 30 min.	Heterotrophic microflora & *L. monocytogenes*	Decreased 1.5 log CFU/g heterotrophic counts. Decreased inoculated *L. monocytogenes* counts by more than 1 log CFU/g. Exposure times of more than 10 min negatively affected red color and rancidity.	[33]

O_3_: Ozone; TVC: Total viable counts; TAMB: Total aerobic mesophilic bacteria; LAB: Lactic acid bacteria; RH, relative humidity; MAP: Modified atmosphere packaging.

**Table 2:** HHP applications to decontaminate meat and meat products.

Meat Type	Treatment Conditions	Storage Conditions	Findings	Reference
Chicken fillets	500 MPa for 10 min.	4 and 12 °C	HHP resulted in the reduction of the pathogen population below the detection limit of the enumeration method (0.48 log CFU/g), irrespective of the inoculum. HHP extended the shelf life of chicken fillets by 6 and 2 days, at 4 and 12 °C, respectively.	[44]
Frozen chicken breast	500 MPa for 1 min and 400 MPa for 5 min.	_	HHP showed inactivation of *Salmonella* at 400 MPa for 5 min and 500 MPa for 1 min.	[45]
Ground chicken meat	350 MPa for 10 min + 0.75% carvacrol.		HHP with 0.60% carvacrol treatment resulted in a >5-log pathogen reduction.	[46]
Ground beef	400 MPa for 15 min at 25, 35, and 45 °C.	4 and −20 °C for up to 5 days	At 25 °C, 5 log reduction in *E. coli* O157:H7 was observed further low-temperature storage serves as the hurdle in its survival and recovery after treatment. HHP showed no effect on the chromatic profile of grounded beef.	[47]
Vacuum-packed ground beef	200 and 400 MPa for 5 min at 25 °C.	_	*L. sakei* is good pressure-resistant lactic acid bacteria used in combination with HHP at 400 MPa and is efficient in controlling pathogenic *E. coli* strains.	[48]
Uncooked ground beef patties	300, 400, and 500 MPa for 5 min.	4 °C for 10 days	HHP combine with *Lactobacillus acidophilus* showed less total aerobic count (3.35 log CFU/g) than untreated (6.74 log CFU/g) beef patties with 0.80 log CFU/mL yeast and mold count. The combined treatment showed a delayed decrease in pH value, inhibited lipid oxidation with better color retention and the highest sensory score.	[49]
Beef patty	400 and 600 MPa for 5 min.	Refrigerated storage for 18 h	An amount of 2 and 4 log CF/mL reductions after 400 and 600 MPa in Shiga toxin-producing *E. coli* O157:H7, respectively. Variations in fat concentration of 10 and 20% did not affect. In contrast, 1% NaCl evident more reduction than 2%, indicating bar protective effect of salt.	[50]
Vacuum-pack ripened mutton patties	200 and 400 MPa for 10 min.	4 °C for 28 days	Significant reduction in total plate count after HHP at both levels, with a significant increase in lightness (L*). Redness (a*), yellowness (b*); hardness, gumminess, and chewiness of patties reduced significantly.	[51]
Beef steak	450 MPa, 600 MPa 1, 3, 6, 10, 15 min.	_	HHP have the potential to allow the production of a convenient and safe product by achieving 5 log definition of pasteurization of beef steak inoculated with *E. coli* 0157:H7.	[52]
Beef slurry	600 MPa for 20 min at 75 °C.	_	Best inactivation of spores of *Clostridium perfringens* in beef slurry was a 2.2 log reduction.	[53]
Beef slurry	600 MPa for 20 min at 75 °C.	_	After HHP, a greater reduction (2.2 log) in *C. perfringens* spores was observed as compared to thermal treatment (no reduction) after 20 min.	[54]
Beef slurry	600 MPa at 70 °C for 20 min.	_	A 4.9 log reduction in *Bacillus cereus* spores after treatment at 70 °C but same temperature thermal processing led to 0.5 log reduction in spore. Increasing HHP temperature from 38 to 70 °C increases the spore inactivation for up to 3 logs.	[55]
Marinated beef (*Longissimus lumborum*)	300, 400, and 600 MPa for 5 min.	Refrigerated storage for 14 days	HHP was proven to provide safe meat along with a sodium reduction in it. Meat marinated with salt and citric acid has no sufficient inactivation of *L. innocua* and *Enterococcus faecium*, while when combine with HHP, a 6 log cycle reduction was observed.	[56]
Beef burgers	300 MPa for 10 min at 9.9 °C and 600 MPa 10 min, 10.2 °C.	_	Mesophilic and psychotropic count remain at the detection limit after HHP at 600 MPa, with no effect on lipid oxidation for at least 6 days.	[57]
Raw meatballs (beef, veal, beef + veal + pork)	400 and 600 MPa for 0 and 18 min.	4 and −12 °C for 18 h	No difference in the extent of inactivation in different species of meat used for meatballs preparation in refrigerated storage (0.9 to 2.9 log CFU/g) as compared to frozen samples (1.0 to 3.0 log CFU/g). A total of 600 MPa requires 1–3 min and 400 MPa requires 9 min for a ≥2.0 log CFU/g reduction.	[58]
Emulsified beef sausages	100–400 MPa for 15 min at 10 °C.	_	HHP proved to be an effective technique to produce microbial safe beef sausages (reduce total viable count equivalent to the sausages having higher salt concentration) with lower salt concentration.	[59]
Dry fermented sausages	600 MPa for 3 min.	4 °C for 4 weeks	Inactivation of *E. coli* O157:H7 in dried fermented sausages was observed to be affected by a_w_. At a_w_ ≤ 0.90, or moisture protein ratio in the range of 1.9–2.3, led to 6.4 log reduction. Further drying reduced to 2.2 log reduction. Recovery of *E. coli* O157:H7 was observed for 1 week of storage but in 2-, 3-, and 4-week storage, no further recovery was observed.	[60]
Pork cooked sausages	600 MPa for 3 min.	4 and 10 °C for 35 days	Cooking of sausages leads to a >6 log reduction in inoculated *L. monocytogenes*. During storage at 4 °C, no significant growth was observed after HHP. But at 10 °C storage, growth remains below the detection limit up to 21 days after the 4.5 log CFU/mL increase in population was observed. No lactic acid bacterial growth was observed till the end of storage.	[61]
Italian salami	600 MPa for 300 s.	_	HHP related microbial inactivation depicts an inverse relation with a_w_. All 20 salami samples showed a 5 log reduction in *Salmonella* after treatment.	[62]
Italian salami	600 MPa for 300 s.	_	An amount of 0.34–4.32 log CFU/g reduction during processing in *L. innocua* was observed which was reduced to 0.48–3.4 log CFU/g after HHP. The efficacy of HHP was associated with a_w_ and higher pH after acidification, drying and seasoning phase.	[63]
Nitrite-free emulsion-type sausage	0.1, 500 MPa for 12 min + 0, 1, 2% vinegar	4 °C for two weeks followed by at 20 °C for three weeks	HHP (500 MPa; four cycles and each for 3 min) + vinegar (1%) reduced vegetative cells and spores of *C. perfringens* by 4.8 and 2.8 log CFU/g, respectively.	[64].
Traditional Portuguese ready-to-eat meat sausage (*Chouriço de carne*)	300 MPa for 5 min at 10 °C + lactic acid bacteria (Pediococcus acidilactici, HA-6111-2) and its bacteriocin (bacHA-6111-2).	Refregrated storage for 60 days.	The hurdle technology (bacteriocin and pressurization) showed a 0.5 log CFU/g decrease in *L. innocua* cells compared to non-treated cells.	[65]
Dry-cured ham	450 MPa for 10 min and 600 MPa for 5 min.	4 °C for 30 days	The efficacy of HHP against *L. monocytogenes* was reduced by low a_w_ values. The changes in HHP-surviving bacteria gene transcription patterns were strain-dependent.	[66]
Cooked ham	400 MPa for 10 min at 17 °C + alginate films containing enterocins.	1 or 6 °C for 2 months	Both antimicrobial packaging and pressurization delayed the growth of *L. monocytogenes* levels below the detection limit (day 90) during 6 °C storage.	[67]

HHP: High hydrostatic pressure; a_w_: Water activity.

**Table 3:** CP applications to decontaminate meat and meat products.

Sample	Experimental Conditions	Target Microbes	Remarks	Citation
Chicken breast meat	In-package DBD-CP: 55–80 kV for 3 min, stored at 4 °C for 24 h or 3 days.	Mesophiles or Psychrophiles	Significant decreases in microbial populations after storage of treated sample for 3 days at 4 °C.	[77]
	In-package CP: 80 kV for 180 s at 25 °C and stored at 4 °C.	Mesophiles, Psychrophiles & *Pseudomonas* spp.	High microbial counts in air packed sample (>6 log CFU/g) than in MAP (<4 log CFU/g) stored for 7 days and 14 days (<6 log CFU/g).	[78]
	32 kHz for 10 min + *Crocus sativus* L., *Allium sativum* L., and *Zataria multiflora Boiss*	*E. coli* & *Staph. aureus*	CP with essential oils reached a satisfactory load below 3.5 log CFU/g. Negative effect on odor, flavor, and overall acceptability.	[79]
	In-package DBD-CP: 100 kV for 1–5 min.	Natural microflora	2 log CFU/g reduction within 5 min in Mesophiles, Psychrotrophic & *Enterobacteriaceae*.	[80]
	DBD-CP: 70 kV for 0–300 s, stored at 4 °C for 5 days.	*Psychrophiles Campylobacter jejuni* & *S. typhimurium*	90% reductions in *Psychrophile*; and 0.5, 0.4, and 0.7 log reductions in *psychrophiles*, *Salmonella*, and *Campylobacter*.	[81]
	In-package CP: 60–80 kV for 60–300 s, stored at 4 °C for 5 days.	*Campylobacter* & *Salmonella*	1.0 log reduction in psychrophiles at 60 kV with 35% O_2_. Also, 60 kV for 60 s treatment with 35% O_2_/60% CO_2_/5% N_2_ reduces microbes and appearance of meat.	[82]
Chicken breast (boiled)	In-package CP: 39 kV for 3.5 min.	*E. coli* O157:H7, *Salmonella*, *L. monocytogenes* & Tulane virus	3.7 log CFU/cube *Salmonella*, 3.9 log 28 CFU/cube *E. coli* O157:H7, 3.5 log CFU/cube *L. monocytogenes*, and 2.2 PFU/cube TV reduction after treatment.	[83]
	In-package DBD-CP: 38.7 kV for 0.3–2.5 min	*Salmonella*	Whey protein coating increased treatment efficacy. An increase in initial inoculum concentration from 3.8 to 5.7 log CFU/sample lead to an increase in D-value increased from 0.2 to 1.3 min with1.7 log CFU/sample (highest) *Salmonella* reduction.	[84]
RTE chicken breast cubes	In package CP: 24 kV for 3 min, stored at 4 °C for 21 days.	Mesophilic aerobic bacteria, *Salmonella*, & Tulane virus	0.7, 1.4 and 1.1 log PFU/cube reduction in mesophilic aerobic bacteria, *Salmonella*, and Tulane virus, respectively.	[85]
Chicken breast patties (ground)	DBD-CP: 70 kV for 180 s at 22 °C, packaged in operating gas: 65% O_2_, 30% CO_2_.	Total plate count	0.9 log reduction after 5-day storage as compared to non-CP treated samples. Rosemary extract prevents lipids oxidation and inhibits microbial growth in CP-processed meat under refrigerated conditions.	[86]
Chicken skin & breast fillet	Plasma jet: Feed gases (argon or air) for exposure times (30–180 s), distances from plasma jet nozzle to sample surface (5–12 mm).	*Campylobacter jejuni*	0.78 to 2.55 and 0.65 to 1.42 log CFU/cm^2^ reductions were observed using argon or air as feed gases, respectively. Argon as a feed gas for a longer time (≥120 s) resulted in the highest reductions.	[76]
Chicken meat	DBD-CP + Paraacetic Acid (PPA 100–200 ppm) 3.5 kHz, 0–30 kV, 0–200 W. for 1–6 min, 2 mm distance.	*S. typhimurium*	2.3 to 5.3 log CFU/cm^2^ reductions with combined treatment in contrast to PAA or CP treatments alone. 4.7 and 5.3 log CFU/cm^2^ was the highest reduction obtained after PAA + CP and CP + PAA, respectively.	[73]
Beef	6 kV and 20 kHz for 30 s–10 min.	*E. coli*	0.9 and 1.82 log CFU/cm^2^ reduction after 2- and 5 min treatments, respectively.	[75]
Pastırma (a dry-cured beef product)	Oxygen (100%), argon (100%) and two oxygen/argon mixtures (25%O_2_/75%Ar and 50%O_2_/50%Ar) for 180 and 300 s.	*L. Monocytogenes*, *Staph. aureus*, total mesophilic aerobic bacteria & yeast–mold	Maximum 0.85 log CFU/cm^2^ and 0.83 log CFU/cm^2^ reduction in *S. aureus* and *L. monocytogenes* counts, respectively. 1.41- and 1.66-log CFU/cm^2^ reduction in total mesophilic aerobic bacteria and yeast–mold counts, respectively.	[28]
Pork loin	DBD-CP: 80 kV for 60–180 s.	Total Aerobic bacteria	53% reduction in total aerobic bacteria showed a significant effect on O_2_ concentration (60%) and time (180 s).	[87]
Pork (fresh & frozen)	Plasma jet: Air 20 kV, 58 kHz, 1.5 A for 0–120 s	*L. monocytogenes* & *E. coli* O157: H7	1.5 log and >1.0 log reduction in *E. coli* O157: H7 and *Listeria monocytogenes*, respectively.	[74]
Ham	2 and 10 kHz, 6.4 or 10 kV for 10–20 min at 22 °C.	*S. typhimurium* & *L. monocytogenes*	1.14 log and 1.02 log reduction in *S. Typhimurium* and *L. monocytogenes* log after 20 min, respectively. CP combined with cold storage for 7–14 days at 8 °C packed under sealed high nitrogen gas flush (70% N_2_, 30% CO_2_) effectively inactivating *S. Typhimurium* (1.84 log) and *L. monocytogenes* (2.55 log).	[88]
RTE ham	In-package DBD-CP: 3.5 kHz, 0–30 kV for 23 °C and stored at 4 °C for 18 h.	*L. monocytogenes*	2 log (CFU/cm^2^) reduction after CP combined with MAP (20% O_2_ + 40% N_2_ + 40% CO_2_) and after 7 days storage at 4 °C cell counts reduced below the detection limit (>6 log reduction).	[89]
	In-package DBD-CP: 3.5 kHz, 0–28 kV for 180 s, and stored for 6 and 24 h at 4 °C.	*L. innocua*	At 4 °C, 1.75 and 1.51 log CFU/cm^2^ reduction on 1% and 3% NaCl ham surface, respectively. At 23 °C, 1.78 and 1.43 log CFU/cm^2^ reduction, respectively.	[90]
RTE mortadella-type sausage	18 kV, 12.5 kHz for 0–120 s, 6 mm distance. Samples were sealed under high nitrogen gas flush (70% N_2_, 30% CO_2_) and stored at 4 °C for 1–21 days.	*E. coli*, *L. monocytogenes* & *S. enterica* serovar *Typhimurium*	The maximum inactivation for *Salmonella* was 0.3 logs. After 120 s CP and storage over 21 days counts for *Listeria* as well as *E. coli* were lower compared to a 30 s treatment (6.58 to 6.25 log and 5.63 to 5 log CFU/g, respectively).	[91]

CP: Cold plasma; DBD: dielectric barrier discharge; RH: relative humidity; MAP: modified atmosphere packaging; PAW: Plasma activated water; RTE: Ready-to-eat.

## Data Availability

Not applicable.
